# Supplementary material for: Shedding Light on the Origin of Egyptian Sheep Breeds by Evolutionary Comparison of Mitochondrial D-Loop
Source: Animals (Basel). 2022 Oct 12;12(20):2738. doi: 10.3390/ani12202738 (PMC9597797; doi:10.3390/ani12202738)
Supplement: Supplementary file 1 [file animals-12-02738-s001.zip › Supplementary figures revised version.pdf]

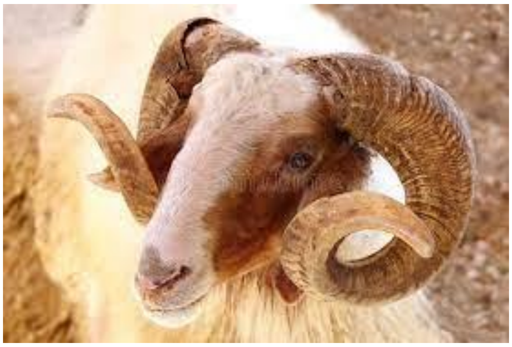

Falllahi (Ram)

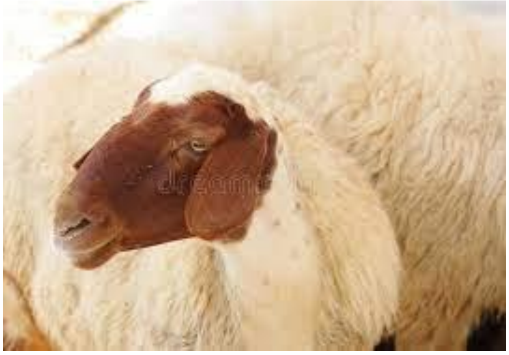

Falllahi (Ewe)

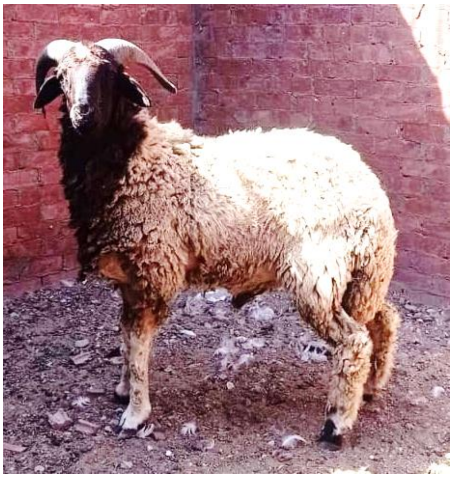

Barki (Ram)

Supplementary figure S1. Pictures of Fallahi (ram and ewe) and Barki (ram) breeds (courtesy of Pr O. Othman and Mr A. Ghazy, respectively).

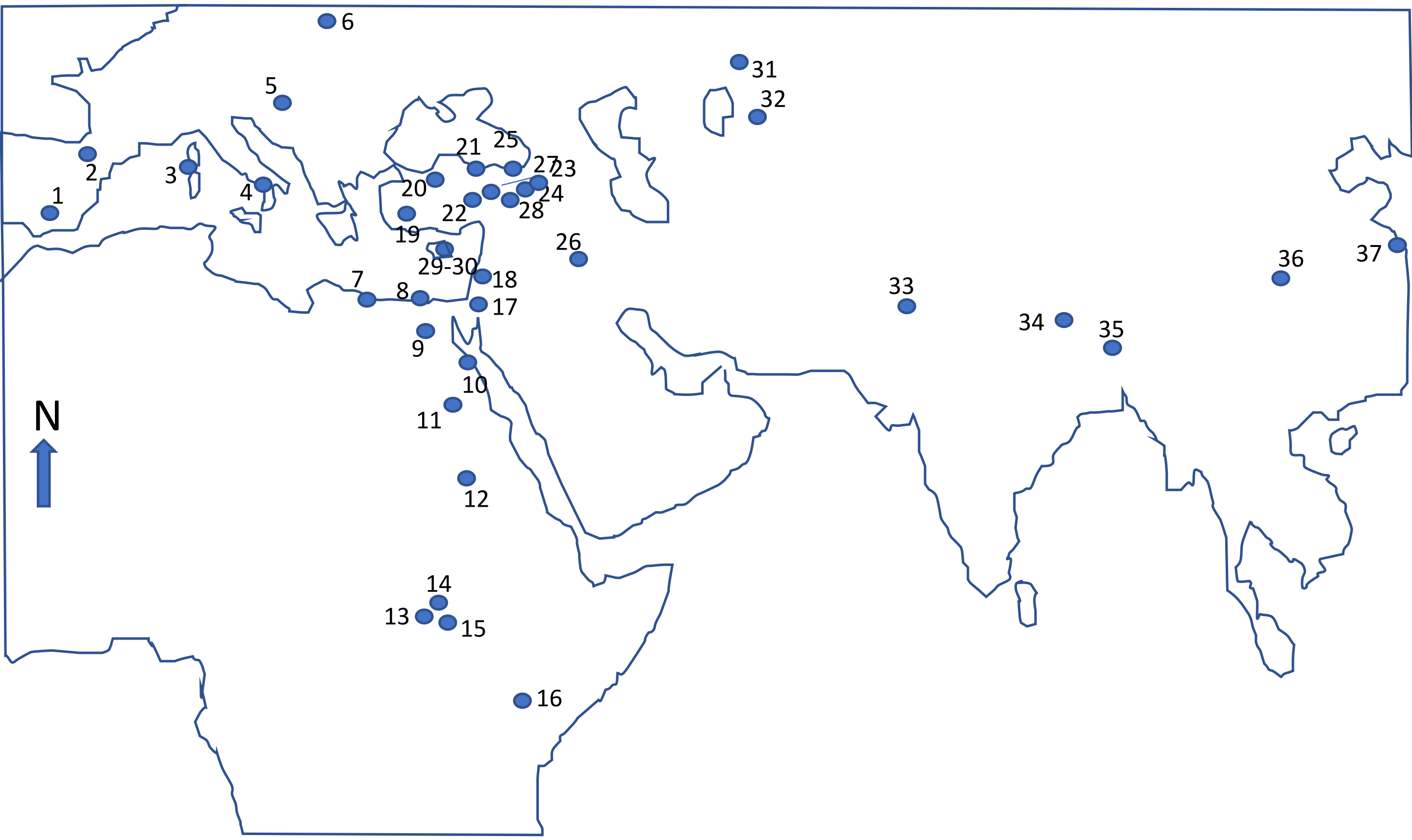

Supplementary figure S2. Localization of breeds.

1: Merino; 2: Latxa Black Face; 3: *Ovis aries musimon*; 4: Italian Laticauda; 5: Waldschaf; 6: Swiniarka; 7: Barki; 8: Rahmani; 9: Ossimi; 10: Fallahi; 11: Saidi; 12 Sohagi; 13; Buzaei; 14: Kabashi; 15: Ahaamda; 16: Red Maasai; 17: Assaf; 18: Awassi; 19: Daglic; 20: Akkaraman; 21: Karayaka; 22: Karakas; 23 Norduz; 24: Morkaraman; 25: Hemsin; 26: Karadi; 27: Tuj; 28: *O. gmelini anatolica*; 29: *O. gmelini ophion*; 30: Cyprus fat-tailed; 31 Edilbai; 32: Lohi; 33: Karakul; 34: Altay; 35: Tibetan; 36: Lanzhou large-tailed; 37: Han large-tailed.

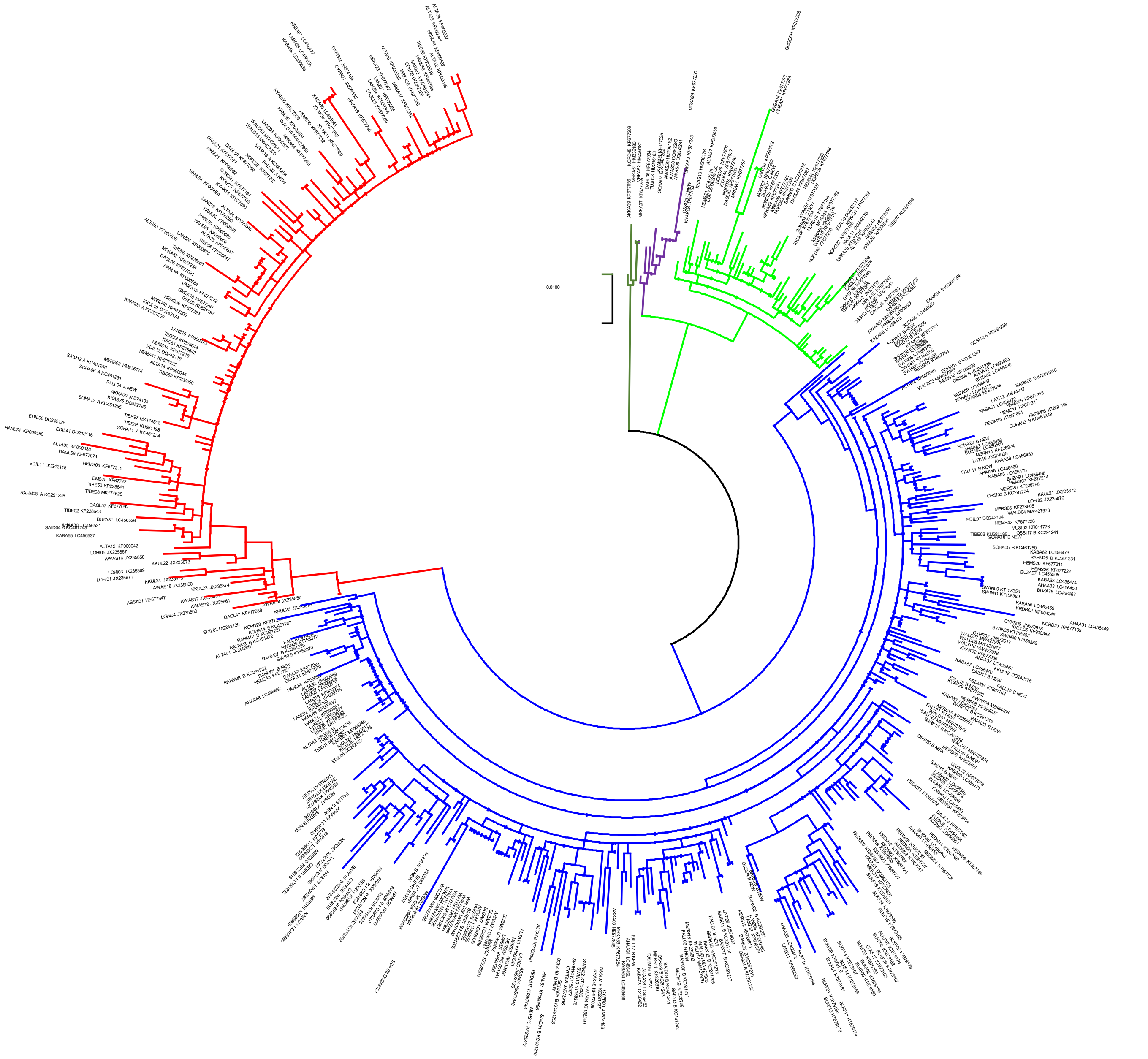

Supplementary figure S3. Phylogenetic tree.

The evolutionary history was inferred by PhyML [28] implemented in Seaview v. 2.4 [29] based on the HKY85 substitution model [30], with a gamma-distribution (Γ) of among-site rate variation (4 discrete categories) [31] and an estimated proportion of invariant sites (0.45). There were 720 homologous positions retained by Gblocks [32] in the final dataset of 467 aligned sequences. The best-scoring maximum likelihood tree has a log-likelihood (LnL) value of -8218.28 and the estimated value of α shape parameter of Γ was 0.44. The tree is drawn to scale, with branch lengths measured in the number of substitutions per site. The drawing was made with MEGA X [27].

The colours of the branches correspond to the haplogroups: red = A, blue = B, light green = C, dark green = D, and violet = E.

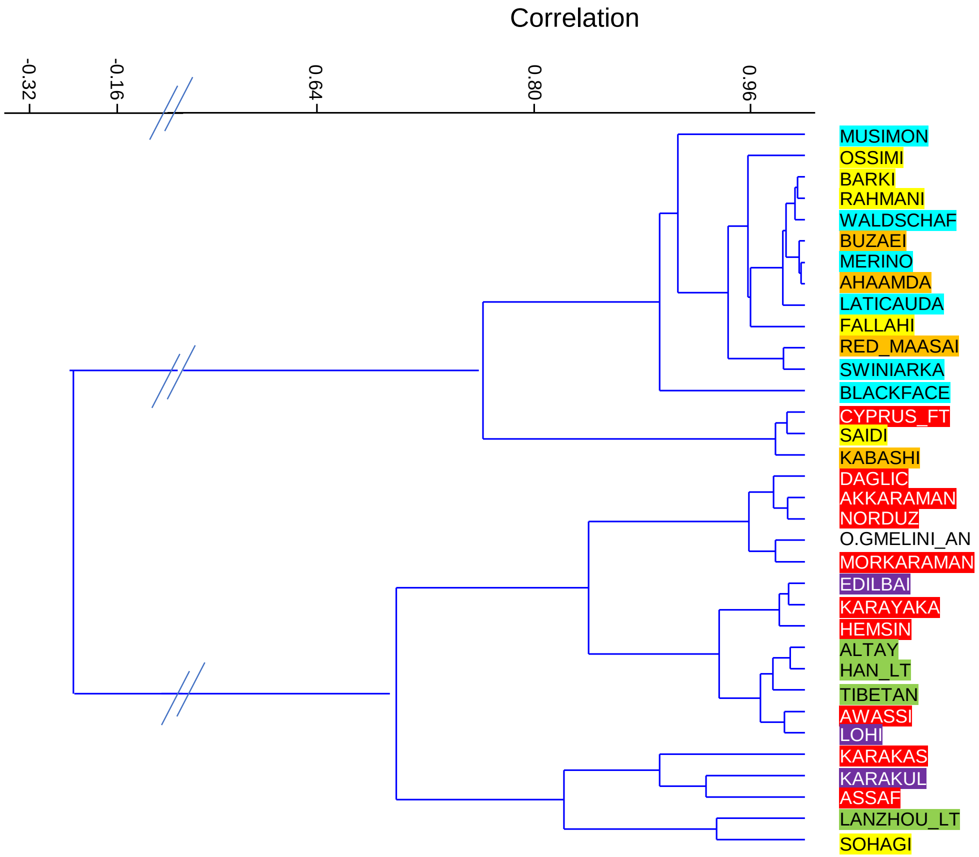

Supplementary figure S4. Cluster analysis of differentiation between breeds.

The *F*st values served to construct the figure, using Pearson correlation as a measure of distance. The background of letters indicates the origin of the breed. Yellow: Egypt; orange: East Tropical Africa; blue: Europe; red: Western Asia; violet: Central Asia; green: Eastern Asia. The drawing was made with PAST v. 4.03 [37].

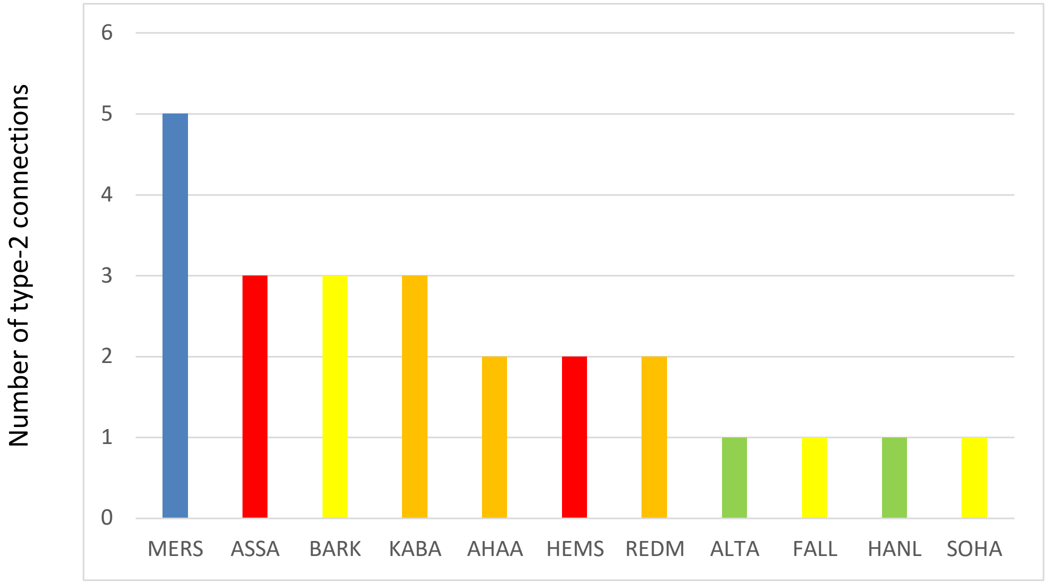

Supplementary figure S5. Type-2 connections between the Italian Laticauda and other breeds.

The colour of each bar indicates the origin of the breed. Yellow: Egypt; orange: East Tropical Africa; blue: Europe; red: Western Asia; green: Eastern Asia. For the abbreviations of breeds, see table 1.
